# Diagnostic Accuracy of the PURE-LAMP Test for Pulmonary Tuberculosis at the County-Level Laboratory in China

**DOI:** 10.1371/journal.pone.0094544

**Published:** 2014-05-01

**Authors:** Xichao Ou, Qiang Li, Hui Xia, Yu Pang, Shengfen Wang, Bing Zhao, Yuanyuan Song, Yang Zhou, Yang Zheng, Zhijian Zhang, Zhiying Zhang, Junchen Li, Haiyan Dong, Jack Zhang, Kai Man Kam, Junying Chi, Shitong Huan, Daniel P. Chin, Yanlin Zhao

**Affiliations:** 1 National Center for Tuberculosis Control and Prevention, Chinese Center for Disease Control and Prevention, Beijing, P. R. China; 2 PATH, China Office, Beijing, China; 3 Stanley Ho Centre for Emerging Infectious Diseases, Faculty of Medicine, Chinese University of Hong Kong, Hong Kong, P. R. China; 4 Bill & Melinda Gates Foundation, China Office, Beijing, China; 5 Respiratory Diseases Department of Nanlou, Chinese People’s Liberation Army General Hospital, Beijing, P. R. China; San Francisco General Hospital, University of California San Francisco, United States of America

## Abstract

**Background:**

Early and effective detection of *Mycobacterium tuberculosis* (MTB), particularly in smear-negative tuberculosis (TB), is a priority for global TB control. Loop-mediated isothermal amplification with a procedure for ultra rapid DNA extraction (PURE-LAMP) can detect TB in sputum samples rapidly and with high sensitivity and specificity. However, the PURE-LAMP test has not been effectively evaluated, especially in resource-limited laboratories. In this study, we evaluated the performance of the PURE-LAMP test for TB detection in TB suspects from two county-level TB dispensaries in China.

**Methodology/Principal Findings:**

From April 2011 to February 2012, patients with suspected TB were continuously enrolled from two county-level TB laboratories in China. Three sputum samples (spot, night, and morning sputum) were collected from each recruited patient. Detection of MTB by PURE-LAMP was compared to a reference standard L-J culture. The results showed that the sensitivity of the PURE-LAMP test based on spot sputum for MTB detection was 70.67%, while the sensitivity of the PURE-LAMP test based on spot sputum for MTB detection in smear positive and culture positive patients and smear negative and culture positive patients was 92.12% and 53.81%, respectively. The specificity of PURE-LAMP based on spot sputum for MTB detection was 98.32%. The sensitivity and specificity of the PURE-LAMP test based on three sputa combination for MTB detection was 88.80% and 96.86%, respectively. The results also showed that the PURE-LAMP test had a significantly lower contamination rate than did solid culture.

**Conclusions/Significance:**

The study suggested that, in peripheral-level TB laboratories in China, the PURE-LAMP test showed high sensitivity and specificity for TB detection in TB suspects, making it a more effective, rapid, and safe method worthy of broader use in the future.

## Introduction

Tuberculosis (TB) remains a major global health problem. In 2012, an estimated 8.6 million people developed TB and 1.3 million died from the disease [1]. Accurate and rapid diagnosis of TB is vitally important in establishing appropriate clinical management and infection control measures [2], [3]. Currently, the most common method for TB diagnosis worldwide is sputum smear microscopy, the sensitivity of which is notoriously poor, particularly in human immunodeficiency virus (HIV)–positive patients [4], [5]. Culture, the gold standard diagnostic method, is highly sensitive but takes between two and six weeks to obtain a result [2]. To address the need for rapid and sensitive diagnosis of TB, a number of nucleic acid amplification assays have been invented [6], [7], [8]; however, they are still not routinely applied in developing countries due to their high cost, complicated procedures, insufficient laboratory facilities, and a shortage of skilled technologists [9], [10], [11].

Loop-mediated isothermal amplification (LAMP) is a novel nucleic acid amplification method that does not require an expensive thermocycler or detection system [12]. TB-LAMP is a new manual TB detection method based on the LAMP platform from Eiken Chemical Company in Japan. TB-LAMP has several features that make it attractive as a diagnostics platform for resource-poor settings: it is fast (40 minutes), isothermal (requiring only a heat block), robust to inhibitors and reaction conditions that usually adversely affect polymerase chain reaction methods, and it generates a result that can be detected with the naked eye [13], [14], [15]. From 2007 to 2010, Eiken Chemical Company and Foundation for Innovative New Diagnostics (FIND) successfully developed a next-generation TB-LAMP kit, which has a procedure for ultra rapid DNA extraction (PURE-LAMP) [16]. PURE-LAMP has increased sensitivity and specificity, thanks to the newly designed primer. Having DNA extraction and amplification in a closed unit significantly reduces the risk of contamination. The PURE-LAMP test consists of three steps, sample preparation (10–20 min), Amplification (40 min), and visual detection of fluorescence light from the reaction tube using UV light (0.5–1 min) [17].

To evaluate the clinical performance of the PURE-LAMP in a peripheral laboratory, we conducted this study in two county-level microscopy centers in Henan Province, China.

## Materials and Methods

### Ethics

The study was approved by the Ethics Committee of the Chinese Center for Disease Control and Prevention. The requirement to obtain individual informed consent was waived by the review board.

### Study Design

The study was carried out in two peripheral microscopy centers (Huojia and Xinxiang counties) in Henan Province, China. From April 2011 to February 2012, new patients with suspected pulmonary TB (cough and expectoration for at least 2 weeks, hemoptysis, and bloody sputum) were consecutively enrolled in the study. Each recruited patient submitted three sputum specimens (spot, night, and morning sputum), the quantity of each specimen should no less than 2 mL (0.1 mL for smear, 1 mL for culture, and 0.06 mL for PURE-LAMP test). Lab technicians from microscopy centers simultaneously tested each specimen by smear microscopy, solid culture, and PURE-LAMP test. China National Tuberculosis Reference Laboratory (NTRL) staff collected all culture-positive strains and performed 16S-23S rDNA ITS sequence analysis for strain identification. Solid culture was used as the reference standard to assess the performance of the PURE-LAMP test by different sputum combinations in TB suspects.

Two microscopy centers did not perform solid culture and PCR test before our study. In order to ensure the testing quality of culture and LAMP, all participating laboratory technicians received training from NTRL. All staff received one week training before undertaking the test. In order to ensure quality of the solid culture, staffs at Huojia and Xinxiang counties have done TB culture for 9 months before field implementation. LAMP pilot was conducted in local sites for 1 month. NTRL has supervised pilot sites in the end of the pilot phase to verify technician’s performance.

### Methods

#### ZN microscopy and L-J culture

The reference standard, quality-assured culture was done by designated technicians who were not aware of the results of other tests. All sputum specimens were subjected to direct smear microscopy using the Ziehl-Neelsen method in accordance with the *Sputum smear Microscopy SOP and Quality Control Manual* [18]. Sputum specimens were processed and inoculated onto acid L-J medium under World Health Organization guidelines [19]. Cultures were inoculated at 37°C and monitored for growth for 8 weeks.

#### PURE-LAMP

The PURE-LAMP test was performed as described previously [17]. Two laboratory technicians from each laboratory were trained as operators and passed proficiency tests after three runs per person using the following procedure: With a wide-bore disposable pipette provided by Eiken, the technicians collected and transferred 60 µL of sputum to a heating tube containing extraction solution. They mixed and incubated the heating tube at 90°C for 5 minutes. Next they attached the heating tube to an adsorbent tube and mixed until all the powder had completely mixed with the solution. They then placed the injection cap on the adsorbent tube and screwed tightly. Next they inserted the nozzle into a reaction tube and transferred 30 µL of solution to the reaction tube. They loaded the reaction tube into the heating block at 67°C for 40 minutes, transferred the reaction tube into the fluorescence detector, and recorded the results.

#### Species identification

All positive cultures underwent 16S-23S rDNA ITS sequencing-based species confirmation by NTRL staff [20].

### Statistical Analysis

The sensitivity, specificity, positive predictive value (PPV), and negative predictive value (NPV) were calculated to assess the performance of the TB-LAMP assay using solid culture as the reference standard. All of the data were analyzed by the China NTRL. All analysis was done with SPSS19.0 software, and P<0.05 was regarded as significant.

## Results

### Study Population

**Figure 1:**
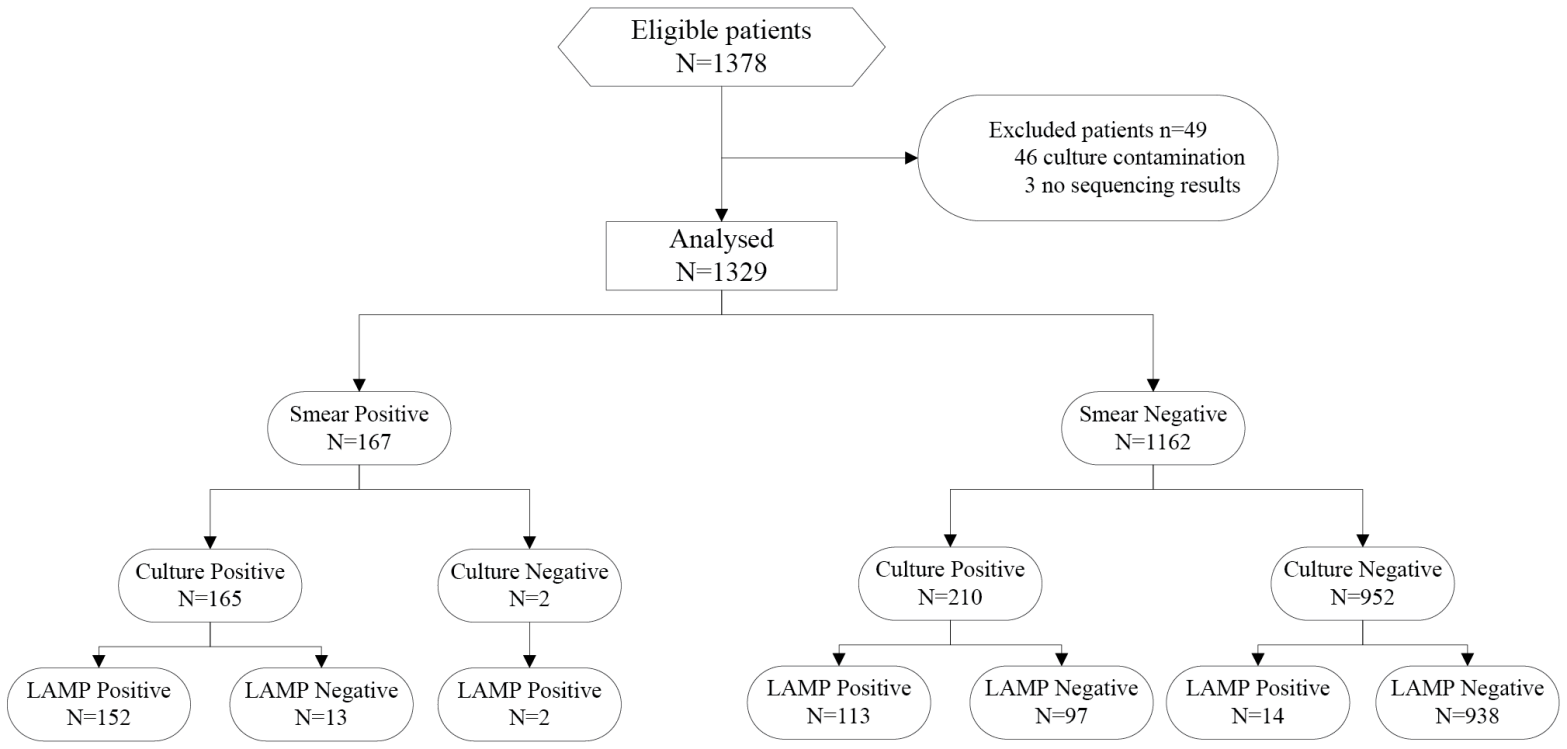
Patient enrollment and testing flow chart.

From April 2011 to February 2012, 1378 eligible TB suspects were enrolled. No patients were excluded from the study because of insufficient specimen to conduct all the 3 tests. For analysis, 46 patients were excluded because of culture contamination, and 3 patients were excluded because of no sequence result, therefore, only 1329 TB suspects were remained for analysis (Figure 1). Of these 1329 TB suspects, 888 were male, 441 were female; 53 of the patients were aged 20 years and under; 165 (12.42%) were smear positive and culture positive TB patients, 210 (15.80%) were smear-negative and culture-positive TB patients, and 954 (71.78%) were culture negative TB patients (Table 1).

**Table 1:** Demographic information for the participants who were analyzed.

Variable	Xinxiang*(N = 902)*	Huojia*(N = 427)*	All patients*(N = 1329)*
	No. (%)	No. (%)	No. (%)
**Age range**			
<20	31 (3.44)	22 (5.15)	53 (3.99)
20–39	369 (40.91)	121 (28.34)	490 (36.87)
40–59	295 (32.71)	153 (35.83)	448 (33.71)
≥60	207 (22.95)	131 (30.68)	338 (25.43)
**Gender**			
Female	280 (31.04)	161 (37.70)	441 (33.18)
Male	622 (68.96)	266 (62.30)	888 (66.82)
**Culture result**			
Smear-positive/culture-positive	117 (12.97)	48 (11.24)	165 (12.42)
Smear-negative/culture-positive	186 (20.62)	24 (5.62)	210 (15.80)
Culture-negative	599 (66.41)	355 (83.14)	954 (71.78)

### Performance of PURE-LAMP Test for MTB Detection Based on Spot Sputum

Among the 1329 analyzed TB suspects, the sensitivity and specificity of PURE-LAMP for MTB detection based on spot sputum were analyzed using solid culture as the reference standard (Table 2, Figure 1). The diagnostic results of 1203 TB suspects (90.52%) were consistent with those by solid culture. The sensitivity of PURE-LAMP based on spot sputum was 70.67%, while the sensitivity of PURE-LAMP based on spot sputum in smear-positive and culture positive TB patients was 92.12%, the sensitivity of PURE-LAMP for MTB detection based on spot sputum in smear negative and culture positive patients was 53.81%. The specificity of PURE-LAMP for MTB detection based on spot sputum was 98.32%.

**Table 2:** Performance of the PURE-LAMP test for TB detection in TB suspects based on spot sputum.

Site and no. of test		Sensitivity		Specificity (no tuberculosis)	PPV	NPV
	All culture positive	Smear positive and culture positive	Smear negative and culture positive			
**Xinxiang**						
Correct–no./total no. (%)	205/303(67.66)	104/117(88.89)	101/186(54.30)	587/599(98.00)	205/217(94.47)	587/685(85.69)
**Huojia**						
Correct–no./total no. (%)	60/72 (83.33)	48/48(100)	12/24 (50.00)	351/355(98.87)	60/64(93.75)	351/363(96.70)
**All patients**						
Correct–no./total no. (%)	265/375(70.67)	152/165(92.12)	113/210(53.81)	938/954(98.32)	265/281(94.31)	938/1048(89.50)

### Performance of PURE-LAMP for MTB Detection Based on Different Sputum and Sputum

Of the 1329 TB suspects analyzed, we calculated the sensitivity and specificity of PURE-LAMP based on different sputum (spot sputum, night sputum or morning sputum) or different sputum combination (spot and night sputum, spot and morning sputum, night and morning sputum, spot and night and morning sputum) (Table 3). The sensitivity and specificity of PURE-LAMP based on the number and combinations of sputum specimens collected were calculated using solid culture as a reference standard (Table 3). The sensitivity of PURE-LAMP for MTB detection based on three sputa is higher than that based on one sputum specimen.

**Table 3:** Performance of PURE-LAMP based on different sputum and different sputum combination.

No. of TB test	Sensitivity	Specificity (No-tuberculosis)	PPV	NPV	Diagnostic accuracy
**3 samples (spot, morning, and night sputum)**					
Correct–no./total no.(%)	333/375(88.80)	924/954(96.86)	333/363(91.74)	924/966(95.65)	1257/1329(94.58)
95% CI	(85.21–91.61)	(95.55–97.79)	(88.45–94.15)	(94.18–96.77)	93.23–95.68
**2 samples**					
**Spot and night sputum**					
Correct–no./total no.(%)	310/375(82.67)	929/954(97.38)	310/335(92.54)	929/994(93.46)	1239/1329(93.23)
95% CI	(78.51–86.16)	(96.16–98.22)	(89.22–94.89)	(91.75–94.84)	(91.75–94.46)
**Spot and morning sputum**					
Correct–no./total no.(%)	309/375(82.40)	928/954(97.27)	309/335(92.24)	928/994(93.36)	1237/1329(93.08)
95% CI	(78.22–85.92)	(96.04–98.13)	(88.87–94.65)	(91.64–94.75)	(91.58–94.32)
**Night and morning sputum**					
Correct–no./total no.(%)	314/375(83.73)	931/954(97.59)	314/337(93.18)	931/992(93.85)	1245/1329(93.68)
95% CI	(79.66–87.12)	(96.41–98.39)	(89.97–95.41)	(92.18–95.18)	(92.24–94.87)
**1 sample**					
**Spot sputum**					
Correct–no./total no.(%)	265/375(70.67)	938/954(98.32)	265/281(94.31)	938/1048(89.50)	1203/1329(90.52)
95% CI	(65.87–75.05)	(97.29–98.97)	(90.95–96.47)	(87.50–91.22)	(88.83–91.98)
**Night sputum**					
Correct–no./total no.(%)	267/375(71.20)	938/954(98.32)	267/283(94.35)	938/1046(89.67)	1205/1329(90.67)
95% CI	(66.42–75.55)	(97.29–98.97)	(91.01–96.49)	(87.68–91.38)	(88.99–92.12)
**Morning sputum**					
Correct-no./total no.(%)	261/375(69.60)	935/954(98.01)	261/280(93.21)	935/1049(89.13)	1196/1329(89.99)
95% CI	(64.76–74.04)	(96.91–98.72)	(89.65–95.61)	(87.10–90.88)	(88.26–91.49)

### Contamination Rate of PURE-LAMP and Culture

In this study, 475 runs of the PURE-LAMP were conducted on new patients with suspected pulmonary TB. Among the 475 runs of PURE-LAMP, only 1 run was contaminated, with a total contamination rate of 0.21%.

A total of 4,129 sputum specimens were collected from the 1,378 patients enrolled at the two dispensaries, with each digested specimen inoculated into two tubes of culture medium. Among the 8,268 cultures, 276 tubes were contaminated, with a total contamination rate of 3.3%.

### Analysis of Discrepant Cases

We analysis the discrepant cases and found that among the 30 culture-negative patients who were diagnosed positive by PURE-LAMP, 2 patients were smear-positive. Of the 42 culture-positive patients who were diagnosed as negative by PURE-LAMP based on three samples, 6 were identified as NTM by sequence, while 36 were identified as MTB by sequence. All the 36 MTB strains were smear-negative and twenty-six of these patients (72.22%) had a colony number of less than 20 for solid culture.

## Discussion

Early diagnosis is important for the control and prevention of TB. With the development of molecular biology, a number of methods have been developed for the rapid detection and diagnosis of TB. LAMP is a new nucleic acid amplification technology first proposed by Notomi in 2000 [12]. The PURE-LAMP test is a new, simple, contamination-resistant kit for the diagnosis of TB.

In this study, the sensitivity of PURE-LAMP based on spot sputum in smear-negative and culture-positive patients was 53.81%, the overall sensitivity of PURE-LAMP was 70.67%, and the specificity of PURE-LAMP was 98.32%, which was similar with that found in other studies [11]. Comparing the sensitivity of PURE-LAMP with that of smear microscopy, the sensitivity of PURE-LAMP was higher than the sensitivity of smear microscopy. This result showed that PURE-LAMP can be used for MTB diagnosis.

Chinese national guidelines have recommended obtaining three sputum specimens from patients with suspected TB [21]. In the present study, we assessed the contribution of each specimen collected to the ultimate diagnosis of MTB for TB suspects and found that the sensitivity of the PURE-LAMP test for MTB detection in TB suspects from three sputa was significantly higher than that in one test of spot sputum. Considering the cost factor, testing three samples may increase the economic burden of patients. Therefore, it is necessary to conduct an analysis of the cost-effectiveness of the PURE-LAMP test with different specimen combinations in the future.

The contamination rate of PURE-LAMP was 0.21% in this study. Two labs in this study were both equipped with three independent and separate working areas, including the storage area for reagents, the pretreatment area, and the amplification and results analysis area. Each room was clearly labeled to avoid confusion when retrieving equipment or materials from different areas. Work benches were disinfected by a 5% sodium hypochlorite solution and exposed to UV light after each testing. Through strict control of contamination and the closed system of the PURE-LAMP test, the risk of contamination was highly reduced.

We also found PURE-LAMP’s reporting time much shorter than that of solid culture: a diagnosis can be completed within two hours. Fluorescence results were examined with the naked eye by lab technicians, and no indeterminate cases were found. China’s NTRL rechecked 20% of the sample results, and the concordance rate was 100%.

In conclusion, the PURE-LAMP test is a new diagnostic technology that can rapidly and accurately detect TB in patients. The test used only 60 µL of sputum each time and was very suitable for pulmonary TB patients, especially for those patients who lacked sputum. Our field study proved that the PURE-LAMP test could be used for screening TB patients in labs in China’s periphery in the future.
